# A Treatise on the Synochus Atrabiliosa, a Contagious Fever, Which Raged at Senegal in the Year 1778, and Proved Fatal to the Greatest Part of the Europeans, and to a Number of the Natives: To Which Is Prefixed, a Journal of the Weather during the Prevalence of That Disease, with Remarks on the Country, Formerly Read at the Royal Society; and Annexed to It, a Short Reflection on the Gum Trade of Senegal, and the Importance of the Place on That Account; Concluding with an Argument Concerning the Bad Consequences Which Must Attend the Present Mode of Sending Convicts to Africa for Soldiers

**Published:** 1784

**Authors:** 


V. *A Treatiſe on the Synochus Atrabilioſa, a contagious fever, which raged at Senegal in the year* 1778, *and proved fatal to the greateſt part of the Europeans, and to a number of the natives: To which is prefixed, a Journal of the weather during the prevalence of that diſeaſe, with remarks on the country, formerly read at the Royal Society; and annexed to it, a ſhort reflection on the Gum trade of Senegal, and the importance of the place on that account; concluding with an argument concerning the bad conſequences which muſt attend the preſent mode of ſending Convicts to Africa for ſoldiers. By* J. P. Schotte,

P. Schotte, *M. D.* 8vo. Murray, *London*, 1782. 169 pages.

THE contagious fever, which is the principal ſubject of this work, and which proved ſo fatal to the garriſon and inhabitants of Senegal, made its appearance in the beginning of Auguſt, 1778, and raged till about the middle of September. The rains in that year ſet in early, and were ſo frequent and heavy, that the iſland became partly overflowed. To this circumſtance Dr. Schotte aſcribes the origin of the fever in queſtion, as it has been obſerved to occur only in thoſe years when the rains are extraordinarily frequent, and of long continuance. — The Europeans, we are told, ſuffered much more by it, in proportion, than the mulattoes; and theſe laſt much more than the blacks. Of ninety-two white people who were on the iſland when it broke out, thirty-three only were left, when the French inveſted the place in January, 1779, and ſeveral of thoſe laboured under a lingering dyſentery, in which the fever had terminated. Of thoſe who died, the greater number were carried off on the 4th or 5th day; but a few died ſuddenly on the 3d, and ſome others lived till the 6th or 7th. They who who ſurvived the 7th day, either recovered, or fell into lingering fluxes, attended with obſtructions in the liver, which ſometimes terminated in ſuppuration; and of which death was ſooner or later the conſequence.

The ſymptoms of this fever are accurately deſcribed by our author, and are in general ſimilar to thoſe which have been uſually obſerved in the malignant fevers of moiſt ſituations in hot climates: theſe ſymptoms, in the greater number of caſes, were, from the very beginning, characteriſtic of great debility; but it ſeems that in ſome patients there were ſigns of inflammatory diatheſis, attended with a hard pulſe. In ſeveral of theſe the diſeaſe was preceded or accompanied by a very painful ophthalmia; and it is remarkable that all thoſe who were afflicted with ſuch an inflammation, died. A continual bilious vomiting uſually prevailed through the whole courſe of the diſeaſe; and in the advanced ſtage of it, moſt patients complained of a burning heat about the pit of the ſtomach, attended with an unquenchable thirſt. The bile vomited up was, at firſt, yellow and liquid; but, as the diſeaſe advanced, became green, brown, and, at laſt, black, and was coagulated in ſmall lumps, which floated in a limpid matter not unlike saliva. The same kinds of limpid matter and coagulated lumps of black bile were likewise evacuated by stool. In those who survived the 3d or 4th day, the skin was filled with petechiæ. Towards the close of the disease, many patients became comatose; the stools came away imperceptibly; the tongue was shrunk up, dry and black; and they were troubled with an almost uninterrupted hiccough. The delirium, we are told, was generally rather mild than violent; and some who were seized with a pain in their throat, and a difficulty of swallowing, died suddenly.

Dr. Schotte considers the definition of Synochus, given by Dr. Cullen in his *Synopsis Nosol. Meth.* as the most applicable to the fever he has described; and as it is customary to annex some epithet to the generic name of a disease, in order to distinguish its particular species from others of the same genus, he has chosen to call it the *Atrabilious Synochus*, on account of the black bile evacuated upwards and downwards, and which was so prevailing and fatal a symptom.

The author enters very fully into the remote causes of this fever, and these he ascribes to the heat heat of the weather, the conſtant uſe of animal food without freſh vegetables, and the brackiſhneſs of the water. In the courſe of this inquiry, we meet with much curious information concerning the climate, diſeaſes, &c. of Senegal.

The continual vomiting which accompanied the diſeaſe, ſeemed to preclude the uſe of remedies, till our author, towards the decline of the epidemic, tried the effects of opium. This leſſened the irritability of the ſtomach and enabled him to adminiſter the bark, which was the medicine on which he ſeems to have placed his chief dependence. He very candidly acknowledges, that the cautions which are to be met with in the moſt celebrated practical writers, concerning the uſe of opium in fevers, prevented him from having more early recourſe to that remedy; and alſo that as the violence of the diſeaſe had much abated before he determined to adminiſter it, he had no opportunity of aſcertaining its good effects by a ſufficient number of trials.—Bliſters, he obſerves, did more harm than good. When the fever was overcome, he found wine, particularly Rheniſh, an excellent medicine.—As prophylactics, he recommends a decoction of ſarſapatilla, and a moderate uſe of wine.

In the courſe of the volume, Dr. Schotte takes occaſion to mention an inſtance of a diſeaſe not known in Europe; with his account of which, as it is curious, we ſhall conclude the preſent article: "The right foot," ſays he " of a black girl had been much in-"flamed for a time, when a boil appeared "afterwards near the inward metatarſal bone, "which breaking, the head (as it was called) "of a Guinea worm made its appearance. In "the mean time another boil near the outward "metatarſal bone opened, and the head of "another Guinea worm ſhewed itſelf. I took "hold of the one and the other, and wound as "much of each as followed eaſily, upon two "ſeparate little ſticks. I continued this work "every day for about three weeks, and then "nothing more would follow by pulling, but "I obſerved, that while I was pulling the one "ſtick, the other was drawn cloſe to the ſkin, "which ſhewed, that what I had wound upon "the two ſticks, were the two ends of one "and the ſame worm. I, therefore, unrolled "the end of the worm from the one ſtick, and "by pulling the other, it re-entered the foot, "in which it made different windings, and "came out at the other boil. The whole

"worm was two yards long, and not fo thick "as the fmalleft chord of a violin, but which "of the ends was the head, if there is any, I "cannot pretend to fay."

